# Expanding boundaries in psychiatry: uncertainty in the context of diagnosis‐seeking and negotiation

**DOI:** 10.1111/1467-9566.13044

**Published:** 2019-12-17

**Authors:** Rhiannon Lane

**Affiliations:** ^1^ School of Healthcare Sciences Cardiff University Cardiff UK

**Keywords:** professional–patient interaction, mental health services, uncertainty, identity, medicalisation, ethnography

## Abstract

Psychiatric diagnosis has become pervasive in modern culture, exerting an increasing influence on notions of personhood, identity practices and forms of self‐governing. The broadening of diagnostic categories and increasing awareness regarding popular diagnostic categories has led to an increased demand for formal diagnosis within clinical encounters. However, there is continuing ‘epistemological uncertainty’ (Fox 2000) surrounding these entities, in part due to their lack of associated clinical biomarkers and their ‘fuzzy boundaries’. Meanwhile, this diagnostic expansion has encountered resistance from those concerned with the alleged ‘over‐pathologisation’ of emotional distress. Drawing upon the concepts of ‘diagnostic cultures’ (Brinkmann 2016) and the ‘looping effects of human kinds’ (Hacking 1995), this article considers some of the competing forces acting upon the contested boundaries of diagnostic categories as they play out within diagnostic interactions. The study involved ethnographic observations of diagnostic encounters within several UK‐based mental health clinics. By focusing on interactions where diagnosis is negotiated, findings illustrate the role played by different kinds of diagnostic uncertainty in shaping these negotiations. It is argued that diagnostic reification plays a key role in the moral categorisation of patients, particularly where there is uncertainty regarding individual diagnostic status.

## Diagnostic cultures: expansion, resistance and uncertainty

Psychiatric diagnosis has become a pervasive presence within modern culture (Brinkmann 2016), exerting an increasing influence on notions of personhood, identity practices and forms of self‐governing. Both modern classification systems such as the DSM–5 (American Psychiatric Association 2013), and the trend towards dimensional models of mental disorder have encouraged the broadening of many psychiatric and neurodevelopmental categories, with increasing numbers receiving diagnoses such as autistic spectrum disorders (Navon and Eyal 2016) and attention deficit hyperactivity disorder (ADHD) (Conrad and Potter 2000). This expansion and increasing lay awareness regarding psychiatric diagnosis has led to growing demands for formal diagnosis (Chan and Sireling 2010) and practices of self‐diagnosis (Brinkmann 2016, Singh 2011), with psychiatric concepts being widely adopted as explanations for personal difficulties and distress. This work expands upon what Brinkmann refers to as ‘diagnostic cultures’ (Brinkmann 2016, 2017), which alludes to the ways in which psychiatric diagnoses are now used – not only to understand and treat mental disorders, but also as ways ‘to interpret, regulate, and mediate various forms of self‐understanding and activity’ (Brinkmann 2017: 170). This article will argue that certain diagnoses are more sought after than others, due in part to an apparent moral hierarchy of diagnosis; in addition, it will be argued that de‐emphasising the ontological uncertainty surrounding more medicalised diagnoses plays a key role in the moral categorisation of patients, adding to the exclusiveness of such categories; this in turn facilitates gatekeeping practices within mental health care by allowing patients to be downgraded.

Research has shown how the growth of collective identities surrounding medical diagnoses are able to shape and challenge professional authority, policy priorities and social identities through the formation of ‘embodied health movements’ (Brown and Zavestoski 2004); this can involve groups of stakeholders such as patients and their family members widening diagnostic boundaries in order to obtain recognition for atypical or ‘milder’ forms of a condition (e.g. Navon and Eyal 2016). This fight for diagnostic validation can be seen in the growth of online communities organised around uncertain and contested diagnoses, which demand recognition of conditions as physical diseases, and offer support which includes advice and strategies on acquiring a diagnosis (Conrad and Stults 2010, Dumit 2006). The meaning of a diagnosis can therefore be partially negotiated by various groups with a stake in medicalising a condition: not only larger organisations such as pharmaceutical companies and advocacy associations, but also individuals with contested diagnoses who have an interest in obtaining access to disability‐related concessions (e.g. sick leave or disability benefits), and who require a narrative to explain their difficulties and differences (Anspach 2011).

Additionally, the marketisation and commodification (Brinkmann 2017) of psychiatric diagnoses has led many individuals to identify with popular categories such as bipolar disorder and autism, reinterpreting and framing their behaviour in the light of (evolving) diagnostic definitions. As illustrated by Hacking's concept of the ‘looping effects of human kinds’ (1995), this reframing can in turn change the way in which such diagnoses are classified (e.g. through practices of diagnostic identification and negotiation which may ultimately contribute to the broadening of diagnostic categories). Psychiatric diagnoses are now heavily featured within mainstream entertainment,^1^ with numerous celebrities now openly discussing their psychiatric labels, often under the guise of reducing stigma. Such accounts of diagnosis are arguably fed into the public arena, offering audiences resources for self‐interpretation, and providing examples of diagnostic performativity which create new ways of enacting disorder (Martin 2007).

The evolving nature of psychiatric classification also reflects the ‘epistemological uncertainty’ (Fox 2000) surrounding these entities, deriving in part from their lack of associated clinical biomarkers, their ‘fuzzy boundaries’ and uncertain aetiologies.^2^ With no definitive way of diagnosing mental disorder, psychiatry remains partly reliant upon personal narratives, making further space for diagnostic negotiation on the part of patients (Healy 2008, Whooley 2010): in addition to the social and political negotiation of diagnosis, there is therefore also often an element of individual negotiation at the microlevel (i.e. within professional‐patient interactions). With the democratisation of medical knowledge, patients approach diagnostic encounters with ‘hybrid diagnostic repertoires’ (Anspach 2011: xiv) which include theories concerning history, diagnosis and prognosis, and ideas about professionals and health services. This has possibly made patients more willing to challenge and dispute diagnosis, and to seek advice outside the doctor‐patient relationship (Lupton 1997). Nevertheless, professionals retain the power to officially assign diagnosis, meaning that if formal diagnosis is required then it must be successfully negotiated.

Given that diagnosis is linked to particular forms of treatment (e.g. through clinical guidelines), these categories also remain heavily implicated in the allocation of clinical resources; for instance, within UK Community Mental Health Team (CMHT) policy guidance, there is a current emphasis on prioritising those with ‘severe and/or enduring mental health problems’, to which both schizophrenia and bipolar disorder are often assumed to belong (e.g. Cardiff and Vale University Health Board 2012: 7, King 2001, McEvoy and Richards 2007). There are therefore likely to be opposing forces acting upon the boundaries of diagnosis at the microlevel, that is from patients seeking recognition for diverse forms of suffering and from those who maintain a stake in narrowing the boundaries of diagnostic categories (e.g. professionals needing to gate‐keep access to more intensive and costly services).

Psychiatric diagnosis has, however, been particularly beset by concerns regarding validity and reliability. Therefore, while recent decades have seen increasing demands for diagnosis and the recognition of new forms of diagnosis, there has – apparently in reaction to this expansion – been a rise in discourses surrounding ‘over‐diagnosis’ (often from psychiatrists themselves), whereby psychiatry has been seen as pathologising ‘normal’ aspects of human life and emotion (e.g. Conrad 2008, Frances 2013, Horwitz 2002, 2011). Critics have also questioned the epistemological certainty surrounding psychiatric diagnosis (through critiques regarding reliability and over‐diagnosis), and the ontological basis of psychiatric categories, that is the validity of the categories and whether they should be conceptualised as disease entities or as reactions to life events.^3^ These uncertainties have likely been a further influence upon diagnostic identificatory practices, illustrating the multiple (often opposing forces) operating upon diagnostic cultures, categories and practices, which undoubtedly impact upon patients who seek out (or resist) diagnostic labels.

Research has also shown that the provision of psychiatric diagnosis is contingent upon a number of social factors, such as resources (Bhugra *et al*. 2011, Estroff 1993), pressure from patients or family members (e.g. Kovshoff *et al*. 2012, Whooley 2010), professional opinions regarding the utility of the diagnosis for a particular patient (Allsopp 2017, Kovshoff *et al*. 2012, Whooley 2010) and previous experiences of professionals leading to the development of ‘intuitive prototypes’ concerning diagnostic categories (Allsopp 2017, see also Light 1980, Luhrmann 2000). This article argues that diagnostic expansion and uncertainty have a fundamental role in shaping diagnostic negotiations and professional categorisation practices within clinical assessments. As places where discussions regarding diagnosis occur, diagnostic assessments are arguably spaces where the boundaries of these categories are being continually negotiated and contested by different actors. The following questions will be addressed: (i) how do cultural ideas concerning diagnosis manifest within and shape diagnostic negotiations within UK mental health clinics? (e.g. notions surrounding over and underdiagnosis, popular depictions of mental disorders, etc.) and (ii) what role do individual, epistemological and ontological uncertainty play in shaping diagnostic negotiations and in the moral categorisation of patients?

## Methods

This article draws on research that formed the basis for a doctoral thesis (Lane 2018) which explored diagnostic identities across pre and post‐diagnostic mental health settings. The study involved ethnographic observations (Hammersley and Atkinson 2007) of diagnostic encounters across three CMHTs and 1‐second opinion psychiatry clinic (PSOC), within the same UK city. Twenty‐six semi‐structured interviews with individuals who had received a psychiatric diagnosis were also carried out; all (except one) were recruited separately from the CMHTs: 11 through a bipolar psychoeducation course and 15 through third sector mental health organisations.

Since psychiatric diagnosis is a complex process which occurs across diverse spaces, a variety of broadly diagnostic assessments were observed: initial screening assessments within CMHTs; psychiatric assessments within CMHTs (both initial and follow‐up) and the PSOC. While initial psychiatry appointments were the most explicitly dedicated to the diagnosis of patients, follow‐up assessments were observed since a definitive diagnosis often takes considerably longer than one session. Initial screening assessments were carried out by two CMHT professionals (usually comprising of community psychiatric nurses, social workers and occupational therapists). I chose to observe initial CMHT screening assessments as professionals had informed me that patients often seek diagnoses within these encounters and diagnosis was therefore frequently discussed (although not formally assigned). These assessments also constitute crucial encounters for patients, since the outcome significantly shapes decisions regarding the allocation of more specialised services. Initial appointments (both screening and psychiatric) within the CMHTs lasted around an hour and involved a fairly thorough exploration of a patient's personal history and psychosocial circumstances. Follow‐up appointments lasted 30 minutes. Second opinion consultations often lasted several hours since they were generally one‐off encounters.

The main corpus of observations consisted of 21 CMHT screening assessments, 6 initial‐psychiatric CMHT appointments, 24 follow‐up CMHT appointments and 5 second opinion assessments (*n* = 56). Two multidisciplinary team screening meetings were also observed. The research was approved by an NHS research ethics committee and by the local trust R & D committee. Information was sent out to patients along with appointment letters to inform them of the study and to allow sufficient time to opt‐out. Before appointments, professionals would double‐check with participants to make sure they had received the study documentation and were happy to participate. I then introduced myself, explained the study and answered any questions. During appointments I made field notes, trying to capture as much dialogue as possible (capturing verbatim quotes where possible) as well as details regarding body language, spatial positioning, tone of voice, etc. Since note‐taking is common practice within such assessments,^4^ my own note‐taking was arguably partly camouflaged. Furthermore, the professionals themselves were used to being observed regularly by placement medical students.

I also observed conversations between staff prior to and following assessments, while professionals wrote up their notes and awaited the next appointment. This backstage behaviour – occurring when patients were not present – was useful since I could observe how professionals categorised patients in ways that were not always communicated to patients themselves. It was thus possible to test my interpretations of professionals’ behaviour during the assessment, by triangulating with their more explicit talk following appointments. It was also illuminating since I witnessed the common practices of informal diagnosing (as noted by Dobransky 2009) and diagnostic denial (i.e. denying a person's claim to a diagnosis), often from professionals without formal rights to assign diagnosis.

A thematic analysis of field notes was conducted using NVivo 10 QAQDAS (Computer Aided Qualitative Data Analysis Software; QSR International, Brisbane, Australia) to facilitate the generation of codes and descriptions. A grounded theorising (Hammersley and Atkinson 2007: 158) approach was taken to the analysis of data, involving a constant interplay between data and theory throughout the research process: ideas were developed to try and explain the data, and the data were then returned to in order to test the fit of these ideas and theories. The analysis was also informed by a social constructionist approach, which focused on the performance of identity within participant ‘accounts’ (Scott and Lyman 1968) and did not take for granted the underlying nature of psychiatric categories and classifications.

Given the word constraints I opted to present observational data from a single detailed case which represents a number of themes that arose across the dataset. This case illustrates in detail the means by which professionals and patients may go about negotiating and contesting diagnosis, and the way in which uncertainty of various kinds are articulated and addressed. Since negotiating a diagnosis is in itself a complex activity which involves a full consideration of a person and their narrative, I felt that the interactional detail and subtle nuances within the process of diagnostic negotiation were important to preserve.

## Negotiating diagnosis and diagnostic upgrading/downgrading

A common pattern in assessments was the tendency for patients to seek out psychiatric diagnoses. This would often elicit sceptical responses from professionals,^5^ who tended to align patients with less exclusive diagnostic categories, such as anxiety and depression (the so‐called ‘common mental disorders’), or informally label them with morally charged diagnoses such as borderline personality disorder (BPD).^6^ This reframing echoes previous research demonstrating the role of implicit and explicit categorisation in determining CMHT gatekeeping decisions (Griffiths 2001); patient selection and rationing were linked to two prominent categories in CMHT documentation at the time: the `seriously mentally ill’ and ‘the worried well’; staff denied individuals access to services by reframing patients’ difficulties as ‘life problems’ as opposed to serious mental illness. Current CMHT guidance similarly suggests focusing resources on those with ‘severe and/or enduring mental disorder’ (Cardiff and Vale University Health Board 2012).

As previous sociological research has shown, healthcare triaging processes and the associated classification of patients are often highly moral exercises (Dingwall and Murray 1983, Hillman 2014, Jeffery 1979). Since the denial of diagnosis can equate to reduced access to services, status and resources, leaving individuals open to accusations of malingering (Clarke and James 2003, Lillrank 2003, Nettleton 2006, Ware 1992), patients are compelled to convincingly enact the ‘sick role’ in order to legitimise their struggles (Glenton 2003, Parsons 1951, Werner and Malterud 2003). Consistent with this, patients within the current study tended to downplay their blameworthiness in various ways, by emphasising the unpredictability and uncontrollability of symptoms, and working to upgrade to a more medicalised diagnosis,^7^ as will be illustrated.

Several professionals in this study spoke of the perception that a diagnosis could appear to absolve patients of responsibility for their problems. However, some diagnoses appear to confer greater protection from such accusations than others, with those perceived to be psychological and functional (as opposed to organic) seen as weakening the legitimacy of suffering (Glenton 2003, Nettleton 2006). Similarly, in the current study, patients appeared to use various strategies to align themselves with more exclusive and medicalised diagnostic categories (most commonly those of bipolar disorder, autism and ADHD), emphasising the characteristics associated with these categories, citing evidence from others to verify diagnostically salient traits and recounting psychiatric family histories. The increasing numbers actively seeking a diagnosis of bipolar disorder in particular (Chan and Sireling 2010) has been noted by psychiatrists, with one psychiatrist in the current study commenting (following an appointment) that this was ‘the first person to *not* want to hear they have bipolar’. By contrast BPD – a diagnosis which is less medicalised (Kendall *et al*. 2009, Sulzer 2015) and has repeatedly been shown to elicit strong negative reactions from mental health professionals (e.g. Dobransky 2009, Lam *et al*. 2016, Markham 2003, Sulzer 2015) – tended to be resisted by patients. As found by Dobransky (2009), staff in the present study appeared to distinguish between those whose behaviour was attributed to illness (e.g. those with psychotic and severe mood disorders) and those considered to have lifestyle/personality/behavioural problems, who elicited more self‐managing guidance and moralising responses from professionals.

### Presenting an authentic diagnostic narrative

The following example concerns ‘Hannah’, a woman in her 30s who attends an initial psychiatry appointment seeking a diagnosis of autism (her partner who accompanies her, mentions before the appointment how long it has taken them to get an assessment for this). However, even before the appointment begins, doubt is cast over the authenticity of Hannah's problems; Dr. A reads aloud from her referral letter that she has had a history of anxiety, depression, attempted suicide and ‘historic abuse’ by her stepfather's brother; her brother has a diagnosis of autism, and Hannah believes she herself has autism. I ask Dr. A whether it is common for patients to come in with an idea of what diagnosis they might have – she says yes, very common because nowadays it is so easy to get information from the Internet and that apparently, you can download diagnostic interviews from I‐tunes (she seems horrified by this). She also comments that people would rather believe they have something to explain their behaviour rather than take responsibility for it themselves. Dr. A. reads out from previous case notes that the patient presented as suicidal and as not making eye contact despite being spotted outside the clinic laughing and eating lunch with her girlfriend. Hannah had claimed to self‐harm by carving names on her skin, although ‘there was no evidence of this’. The notes already cast doubt over the legitimacy of her accounts, and much of the assessment is characterised by professional scepticism. Here is an extract from my field notes during this encounter:

Dr. A begins by exploring Hannah's depression and anxiety, asking what makes her anxious. She replies that noises disturb and overwhelm her, as well as socialising and going out, e.g. making small talk and interacting with people as opposed to crowds per se. She also finds talking on the phone difficult. Dr. A asks if she had phoned up to cancel todays appointment would Hannah have answered? she replies no she would let it go to voicemail. Dr. A says it is important to answer these types of calls – she would never leave a message for a patient as you don't know who might hear it – so it is important to answer phone calls as you don't know what opportunities you might miss.

Dr. A appears to be testing the severity of Hannah's anxiety when asking whether she would answer the phone: however, when Hannah confirms the seriousness of her problems by indicating that she would not answer, her behaviour is framed as a harmful choice, rather than as a sign of disorder. The attributes emphasised by Hannah match closely to common symptoms of autism, such as difficulties with social interaction, and unusual sensitivity to sensory stimuli (American Psychiatric Association 2013), also noted by Hacking (2009a) to be a common feature of autism autobiographies. Similarly, when asked what she enjoys doing Hannah says only that she loves playing with Lego, which she collects. She tells how when she was a child she needed to collect toys as opposed to wanting them to play with, suggesting another feature consistent with autism: ‘restricted, fixated patterns of interests that are abnormal in intensity or focus’ (American Psychiatric Association 2013).

When asked about family background, she provides further evidence to indicate her compatibility with autism by emphasising her resemblance to her brother, who has been diagnosed with autism, stating that they get along really well as they both ‘think in the same way and are really similar’. Hannah draws upon understandings of the genetic inheritance of mental disorders as further evidence:

She says her father definitely had ‘something wrong with him’, although undiagnosed as he did not talk about his emotions. Dr. A asks about her mother and she says her mother also believes herself to have autism as well as problems with depression (and severe postnatal depression). It was her mum who suggested that Hannah might have autism. She also mentions a cousin on her father's side who has a diagnosis of autism, further establishing a family link on both sides of the family.

Professionals consistently asked about family histories of psychiatric problems in the assessments, and patients demonstrated awareness of the diagnostic significance of genetics, by readily divulging details of family members’ psychiatric problems or general eccentricities. In the above example, Hannah suggests that her dad had something undiagnosed, which further strengthens her implied genetic predisposition to mental health problems.

### Atypical diagnostic presentations and reframing diagnostic boundaries

Despite Hannah's establishing of a family link to autism, her attempts are undermined by her atypical presentation and high‐functioning status, as will be shown in the following extracts. She possesses characteristics which undermine her compatibility with autism (e.g. managing to hold down a job and interact successfully with others; her autism wasn't picked up as a child etc.). As such she must work to reframe classic notions of autism in order to incorporate her particular symptoms, by emphasising the hidden nature of her symptoms, and her use of adaptive strategies which allowed her to hide her symptoms.

Dr. A tells Hannah that one of the criteria for an autism diagnosis is that the associated symptoms must have been present before the age of three; she would therefore need Hannah's mother to be present in order to do a formal diagnostic assessment for autism. She continues her pre‐assessment by asking Hannah if she has always been the way she is now

Hannah replies yes immediately, stating that she has always felt different: ‘like an alien’. She describes struggling to fit in, thinking differently to others and difficulties seeing from others’ viewpoints. In pre‐school teachers would tell her to make friends but she was unable to relate to other children and would just be doing her own thing, that she always got on better with adults as a child. She suggests that her teachers probably just thought she was shy, but it was that she was different. She adds that she learnt to cope with this and fit in by putting on characters, playing a game called ‘let's try to be normal’ where she would pretend to be a normal person. She says school reports would always mention how she needed to make more friends. Dr. A asks if she still has these reports (presumably to bring to the official diagnostic interview). Hannah explains that a lot of stuff at her nans would have been thrown out after her death, but that she would see what she could find.

In using the alien metaphor to describe her feelings of difference, Hannah is drawing upon a common trope found in some autism communities (Hacking 2009b). By stressing her active use of coping skills in hiding her difference, she justifies the lack of obvious autism traits; those traits which were visible (i.e. lack of social interaction) are depicted as having been misinterpreted as signifying more common difficulties (i.e. shyness). She emphasises her proactive use of coping skills again when asked about secondary school, explaining that she had friends because she realised this would be necessary to avoid being bullied: her apparent normality is explained by her self‐preservation instinct. Further on in the appointment, Dr. A expresses further surprise at Hannah's career success given her interpersonal difficulties:

Dr. A asks why Hannah is unable to make eye contact with her today (joking about the center not being very nice to look at and about herself being ‘scary’) even though she has done all these jobs where making eye contact and body language etc. are all very important. Hannah nods as if she understands what Dr. A is getting at. She explains that this is due to the ‘characters’ that she puts on: in work she adopts these characters as she knows this is appropriate, whereas in this situation she thinks it is better for the doctor to see her as she really is; that she is not putting on any act and is acting naturally. She adds that her colleagues might look at her now and say: ‘that's not the real Hannah – the way she acts in work is the real Hannah’ – but that's not true.

Hannah fights to establish the authenticity of her autism symptoms by presenting her ‘normal’ behaviour as reflecting an inauthentic version of herself; her ‘autistic’ self is presented as the authentic version. The term ‘characters’ emphasises the artificiality of her attempts to adopt normal mannerisms. This presentation of an inauthentic self establishes external behaviour as an unreliable diagnostic measure, since it does not accurately reflect the inner authentic self on which diagnosis should be based. This notion is premised on an ‘essentialist’ model of diagnosis, whereby disorders are defined by their ‘true’ underlying nature and reified as natural kinds (Zachar and Kendler 2007). The ontological status of autism as an entity goes unquestioned: an individual may ‘have’ this disorder but learn how to hide it.

### Justifying diagnostic stake

This contrast between an authentic and inauthentic self is further described when Dr. A asks Hannah whether she is still on sick leave from her current job:

Hannah affirms that she finds her current job really stressful as she is in a noisy office environment where you have to make pointless small talk with people which she finds really difficult. She has gotten to the point where it is just too much putting on the act and that her ‘real self is just spilling out’. It emerges later that she has in fact handed her notice in and she explains how she couldn't carry on making herself miserable anymore by working in this environment. She had requested to work from home some of the time to make things easier, but this was refused and she handed her notice in.

Hannah's unemployed status and lack of leniency from her employers may undermine the authenticity of her account, since it reveals a morally problematic stake in obtaining an autism diagnosis. Dr. A. addresses this by asking Hannah explicitly what she hopes to gain by obtaining a diagnosis, compelling her to provide a more morally acceptable motivation, which draw upon notions of identity fit, self‐awareness and self‐management:

Dr. A. states that she is uncertain about Hannah's diagnostic status, and further that there is a limit to how much they can help (with autism). She asks what Hannah is hoping to get from the diagnosis and why is it important to her, since having the diagnosis might not make things much better, as there is not much help available for this (i.e. autism). Hannah says that she would find it helpful just in understanding herself, because if she knows what's wrong with her she can research it and know what strategies to put in place to help herself. She explains how she knows she has depression and anxiety etc. but that with autism it just fits; she has felt for a long time that she has it and when her brother was diagnosed she read about it and thought ‘that's me: that describes me exactly’. She also says she feels like she is ‘wearing clothes that don't fit’ and that ‘the jigsaw pieces don't fit together’. She wants ‘clothes that fit’ (i.e. appropriate diagnosis). Dr. A nods understandingly here.

The issue at stake in the above extract is not only the patient's authenticity and problematic fit with the diagnostic category but also the utility of receiving a diagnosis, suggesting a model of disorder which emphasises usefulness rather than any underlying reality. However, further on, when Dr. A. explains that she will see Hannah for a formal diagnostic interview, autism is described less as a practical category, and more as an objective entity: an essentialist as opposed to a nominalist approach to psychiatric diagnosis (Zachar and Kendler 2007). With such an essentialist approach it is not sufficient to demonstrate the presence of traits which might require professional attention and increase vulnerability to other mental health problems (e.g. difficulties adapting to change, social interaction and abnormal repetitive behaviour); in order to receive a diagnosis and its associated benefits, there is a need to achieve full category status by proving the underlying reality of disorder (e.g. by drawing on ‘objective’ evidence from parents and teachers about the presence of traits prior to the age of three).

### Reframing as functional disorder

At the end of the session Dr. A explains that she will see Hannah for a proper diagnostic session with her mother (a ‘DISCO’ – diagnostic interview for social and communication disorders), although she expresses doubt as to whether Hannah ‘has’ autism or not. She describes Hannah as ‘a bit of a mixed bag’ – she has some of the traits but some she doesn't have and that she has also had to deal with many traumatic and difficult situations and has had to come up with strategies for dealing with these, and it seems to be implied that this reaction to traumas may better explain her current difficulties. Dr. A. mentions that Hannah doesn't fit typical autism in some ways, e.g. she is not good at maths but is good at English, explaining how typically people with autism do not understand metaphor or poetry – they may like the rules of grammar, but they tend to like maths because it is a matter of following the rules.

Here it seems that the incidence of childhood trauma is taken as evidence against the presence of autism, consistent with the dominant view of autism as organic (as opposed to functional) in nature. It also represents an ‘entity’ perspective, emphasising uniformity as opposed to unique individual expressions of disorder (Zachar and Kendler 2007).^8^ Past trauma was also used to provide evidence against developmental and psychotic disorders in other appointments. While aetiological information may be useful for professionals in forming an overall picture of a patient, using such information as evidence *against* a particular diagnosis may disadvantage (i.e. make diagnosis a more difficult process for) those with documented trauma.

As argued by Navon and Eyal (2016) and consistent with Ian Hacking's ‘looping effect’ (1995), it is possible that patients’ attempts to match their own symptomatology to a particular diagnosis might result in the broadening of the categories in question. For instance, Hannah presenting the argument that she has learnt to disguise her symptoms encourages the consideration of invisible and internal symptoms in diagnostic decision‐making, potentially leading to the widening of the diagnostic category to include those with unusual or less extreme symptoms. Such tendencies may be shaped by popular discourses concerning the underdiagnosis of certain populations, for example, the alleged underdiagnosis of autism in women has been commonly presented in both popular and academic mediums in recent years (e.g. Cheslack‐Postava and Jordan‐Young 2011).^9^ Because of individuals like Hannah fighting for a diagnosis, more of those who are ‘a mixed bag’ and less ‘typical’ diagnostically, may end up widening and changing the diagnostic category in question.^10^

After Hannah (relatively successfully) justifies both her lack of obvious autism symptoms and her desire for a diagnosis, Dr. A ends by downplaying the importance of obtaining a diagnosis by stressing the primacy of Hannah's anxiety issues in her future self‐management:

Dr. A states that even if Hannah receives an autism diagnosis – she still thinks that the major thing for her will be in learning how to ‘manage her anxiety’, and to not be hard on herself: we cannot all be the same – it is just about doing what you have already been doing – trying to act in the appropriate ways even if you feel different and accepting that you are different etc. She recommends a local cognitive behavioural therapy (CBT) course on managing anxiety called ‘living life to the full’, which Hannah has already been on and found somewhat helpful. She also recommends an anxiety management app from Bristol University and talks about the helpfulness of breathing exercises.

The conclusion of this encounter is that Hannah achieves a provisional diagnostic status: her strategies have obliged Dr. A to investigate her diagnosis further. However, perhaps due to her uncertain diagnostic status, her difficulties are subject to reframing and downgrading as functional and anxiety‐related, which – as per the advice on breathing techniques and CBT – is something that she must learn to self‐manage. Hannah is still subject to downgrading since the anxiety is given primacy above the autism associated traits. This downgrading to emphasise anxiety/stress was a common feature of CMHT assessments, allowing professionals to highlight patients’ own agency in managing their mental health, and to steer them towards less intensive services and self‐management such as primary mental health courses, self‐help ‘apps’, mindfulness and ‘bibliotherapy’.

The ontological uncertainty surrounding psychiatric diagnoses is not voiced within the appointment even though the lack of clear boundaries between diagnostic categories would arguably be potentially helpful information in Hannah's case. This reification and adherence to categorical understandings of disorder was common across assessments and – when combined with individual diagnostic uncertainty – appeared to facilitate downgraded of patients’ difficulties to a less medicalised category (i.e. ‘mild or common mental disorder’ rather than ‘severe or enduring mental disorder’), which allows the responsibilisation of patients for their own difficulties. Consultations featuring diagnostic uncertainty – such as Hannah's, contrasted sharply with assessments where individuals were given a clear diagnosis (particularly those diagnosed with a more severe/exclusive category), who were usually responded to in a sympathetic manner. This downgrading could also have practical implications for their access to services both within and outside of the clinic, as suggested by Hannah's (unsuccessful) attempt to obtain flexible working arrangements at work.

### A moral hierarchy of diagnosis

While the moral downgrading evidenced by Hannah's case may be fairly subtle, a more explicit relationship between demedicalisation and moral downgrading was evident in cases involving suspected personality disorder, where professionals would frequently refer to the responsibility of patients for their own difficulties, echoing previous findings regarding BPD in particular (e.g. Bonnington and Rose 2014, Dobransky 2009, Sulzer 2015). However, the current study suggests a moral hierarchy of diagnosis within psychiatry, whereby diagnoses associated with psychosis (such as bipolar disorder and schizophrenia) hold greater currency than personality disorder or common mental problems (such as anxiety‐related disorders and depression). This echoes previous findings on the prestige rankings of disability, where less medicalised diagnoses were ranked lower, and where common mental disorders such as depression and anxiety ranked particularly low (Grue *et al*. 2015).

In the following interview extract, Cerys, a participant with a diagnosis of recurrent depression, describes her reaction to the suggestion that she falls short of meeting the criteria for bipolar disorder, having sought a second opinion from a private psychiatrist:

Cerys: Well the other diagnosis that was in the mix since last November was … well, it was at the same time as the dissociation, saying that they were ruling out bipolar because although I had characteristics of mood instability, I don't become elated enough to meet the markers. So, with the dissociation was a suggestion that I have … untrue bipolar, and I was thinking ‘god I can't even have proper bipolar’, you know the experience of the highs and the lows because I don't meet the high.

(….)

I: So how did you feel when you heard that?

Cerys: Well it's a bit like failure isn't it – it's a bit like you can't even have the right diagnosis.

Cerys's comment that not achieving bipolar status feels like a failure suggests not only a moral hierarchy of psychiatric diagnoses, but also the implications of a sub‐threshold diagnostic status which is arguably exacerbated by the dominance of categorical understandings of diagnosis. Once again, the diagnostic uncertainty becomes an individual matter, as Cerys ‘fails’ to meet the threshold for bipolar disorder and is placed into the arguably lesser category of ‘untrue bipolar’; the locus of uncertainty does not surround the diagnosis itself and its boundaries. Arguably, if dimensional notions of diagnosis were more dominant, this marginal status would be more normalised for patients.

## Discussion

By focusing on interactions where diagnosis is negotiated, this article contributes to the sociology of diagnosis by illustrating the opposing forces acting upon the boundaries of diagnostic categories within clinical interactions. It also explores the role played by different types of uncertainty in shaping diagnostic interactions which have significant psychosocial consequences for patients; in particular it focuses upon the implications of individual diagnostic uncertainty when combined with a de‐emphasis upon ontological uncertainty regarding the underlying nature of psychiatric diagnosis. It is argued that the diagnostic reification and silence regarding ontological uncertainty plays a key role in the moral categorisation of patients, facilitating the downgrading of patients, and allowing them to be more easily redirected towards less intensive services. Paradoxically, epistemological uncertainty arguably facilitates both the contesting of sought‐after diagnoses (such as bipolar disorder and autism) and the enabling of informal diagnostic practices by professionals.

As shown by previous sociological research, diagnosis demonstrates itself to be a moral business, with a moral hierarchy of diagnosis within CMHT settings which many patients displayed an awareness of. This is illustrated by patients’ frequent attempts to negotiate more validating diagnoses, using strategies to align themselves with more exclusive categories and drawing upon popular discourses regarding the underdiagnosis of certain populations. It is unlikely that these are conscious or dishonest strategies; rather, they illustrate how ‘looping effects of human kinds’ (Hacking 1995) occur in practice, with patients coming to identify with particular labels, contributing to the broadening of diagnostic boundaries and changing the meanings associated with diagnostic categories (e.g. through the inclusion of atypical or less extreme symptoms). Contrastingly, professionals tended to downgrade patients’ problems, withholding more exclusive and medicalised diagnoses, and consequently support.

In focusing on community‐based services – where people mostly *choose* to attend appointments – this study likely portrays a partial view of diagnostic cultures and practices in psychiatry. There are clearly also many who actively resist medicalisation; however, such individuals are less likely to attend appointments and were therefore infrequent in this study. It is also not the intention here to suggest that all patients seeking out a medicalised diagnosis should receive one: there are clear downsides to receiving a psychiatric diagnosis, and many would argue the merits of withholding a formal diagnosis from patients, such as in encouraging a stronger sense of agency. However, while critics of psychiatry have tended to focus upon the stigmatising consequences of diagnostic labelling, this study suggests that attention must also be given to the consequences of withholding diagnosis, particularly for individuals who have a liminal or uncertain status. The demedicalisation involved can manifest as ‘volitional stigma’ (Easter 2012), whereby behaviour is interpreted as an ongoing voluntary choice rather than stemming from mental illness. Patients may seek out more medicalised categories in order to offset such moralising reactions. Thus, although professionals may find diagnosis‐seeking behaviour difficult (Chan and Sireling 2010), practices of diagnostic downgrading may inadvertently prompt this tendency in patients, who will be further driven to upgrade their medical status. This tendency is perhaps likely to be particularly evident within CMHTs, where resources are particularly constrained within the context of economic austerity.

The perpetuation of categorical certainty combined with an emphasis upon individual diagnostic uncertainty allows patients to be clearly excluded from membership of more exclusive and sought‐after categories (such as bipolar disorder and autism), contributing to this cycle of diagnosis seeking and downgrading. This allows more intensive services (e.g. secondary mental health services) to be withheld, and for moral responsibility to be re‐directed onto patients themselves. It also places the source of the uncertainty within the individual themselves, rather than within medicine or the mental health system. The categorical understanding of diagnosis – whereby individuals are considered to either ‘have’ or not have a diagnosis – arguably disadvantages those such as Hannah and Cerys, who exhibit atypical symptoms of a particular category, or for those whose complex presentations cannot be reduced to one category. There will be implications for their ability to access important services and resources, and for their sense of self.

Less reification of psychiatric categories (e.g. by discussing concepts such as diagnostic continua, overlap and heterogeneity) might arguably ease the suffering of patients whose diagnosis is uncertain. Scholars have highlighted both the potential benefits of emphasising uncertainty within clinical communication (Buetow 2011, Gordon *et al*. 2000) as well as the harms associated with categorical reification and denial regarding ontological uncertainty (Pickersgill 2009). Although evidence suggests that professionals are often aware of the imperfect knowledge regarding diagnostic categories (e.g. Rafalovich 2005), this study suggests that ontological uncertainty is not routinely communicated with patients, with discussions tending to centre upon whether patients ‘had’ or did not ‘have’ a particular diagnosis. This tendency for clinicians to de‐emphasise uncertainty has been noted by several sociologists (e.g. Atkinson 1984, Buetow 2011, Fox 2000, Katz 1984). However, findings here suggest that it is ontological uncertainty in particular which often remains unarticulated, while uncertainty regarding individual diagnosis *was* expressed. Given that diagnostic uncertainty is a common feature within psychiatry, a move towards dimensional models which emphasise severity, incorporate heterogeneity and emphasise individual symptoms as opposed to categorical status, may be more appropriate within clinical communication, particularly for those on the boundaries of a diagnosis.

## Notes

1 As pointed out by Hollin (2017), autism fiction has evolved into a recognised genre.

2 While there are some exceptions to this, biomarkers are not generally used for diagnostic purposes in psychiatry.

3 Schizophrenia, ADHD (e.g. Rafalovich 2005) and personality disorder have faced particular criticism regarding their validity.

4 Psychiatrists would take notes during their consultations, and during screening assessments, one professional would generally take thorough notes, while the other would ask questions.

5 At times, following appointments – professionals explicitly accused patients of seeking a diagnosis for the purpose of claiming benefits.

6 Diagnoses of PD were often assigned informally during professional discussions and not communicated to patients. This was in the vast majority of occasions referring to BPD and (in all but one case) applied to female patients.

7 For instance, on several occasions patients with a diagnosis of BPD actively sought out more medicalised diagnoses such as bipolar disorder. Three patients were observed *explicitly* looking to change their BPD diagnosis to bipolar disorder; several interview participants also explicitly described having gone through this process. Several patients (such as Hannah) also sought diagnoses of developmental disorders as opposed to anxiety/depression.

8 ‘Entity’ perspectives have been dominant in psychiatry since DSM–3, while unique individual expressions of disorder were advocated by DSM–1 (Zachar and Kendler 2007).

9 This argument has also been made regarding ADHD.

10 This broadening of a diagnostic category has also occurred with bipolar spectrum disorders (e.g. Angst and Gamma 2002).
